# Medical School Interview Preference in the Post-COVID-19 Era: A Single Institution Experience

**DOI:** 10.7759/cureus.51042

**Published:** 2023-12-24

**Authors:** James C Mamaril-Davis, Mary Nguyen, Jonathan Yasmeh, Emily Leyva, Ran Li, Hongyi Wang, Tejal Parikh

**Affiliations:** 1 College of Medicine, The University of Arizona College of Medicine - Tucson, Tucson, USA; 2 College of Medicine, University of Arizona College of Medicine - Tucson, Tucson, USA; 3 Department of Educational Psychology, University of Arizona College of Medicine - Tucson, Tucson, USA; 4 Department of Disabilities and Psychoeducational Studies, University of Arizona College of Medicine - Tucson, Tucson, USA

**Keywords:** virtual interviews, admission process, medical school interview, medical school applicant, covid-19

## Abstract

Background: The coronavirus disease 2019 (COVID-19) pandemic presented unforeseen obstacles to prospective medical students such as Medical College of Admission Test (MCAT) scheduling postponements and technical challenges during virtual interviews. Students were also faced with difficult decisions post-submission such as having to choose a program without ever visiting a school in person. The primary objective of the present study is to assess the changes in medical school interview preferences and experiences in the post-COVID-19 era.

Methods: A retrospective survey of the class of 2024 (in-person interview group) and class of 2025 (virtual interview group) at an allopathic medical school was conducted in the Fall of 2021 via the Qualtrics XM online survey software (Qualtrics, Provo, UT, USA).

Results: There were 195 survey respondents: 77 students from the in-person interview group and 89 students from the virtual group. More students in the virtual cohort had to reschedule their MCAT compared to the in-person cohort (56.1% versus 14.3%; p<0.001). The in-person group had higher travel-related expenses (>$500) compared to the group who interviewed virtually (65.1% versus 2.4%; p<0.001). More students from the in-person cohort preferred the in-person interview format compared to the virtual cohort (85.7% versus 22.5%; p<0.001). Lastly, 87% of the in-person group and 24.7% of the virtual group felt they were able to gather a clear impression of the atmosphere and culture of a school from the interview trail alone (p<0.001).

Conclusion: Matriculated medical students at an allopathic medical school who applied during the COVID-19 pandemic had more pre-application hurdles when compared to the cohort who applied just prior to the pandemic. Students who primarily had virtual interviews during the pandemic had less travel-related costs but felt more limited in their experience of a school’s culture and ability to establish rapport with interviewers. Despite this, however, the virtual group still expressed a preference for virtual interviews.

## Introduction

The coronavirus disease 2019 (COVID-19) pandemic had irrevocable effects on healthcare organization and society at large [1]. The effects, positive and negative, on medical school admissions were no different. The pandemic ushered in new obstacles and considerations for candidates applying to medical school such as Medical College of Admission Test (MCAT) postponements, financial barriers, and disruptions in clinical, volunteer, and/or research experiences. From the start of the pandemic in March 2020, potential candidates studying for the MCAT faced both changes to the format of the examination and scheduling interruptions due to test site closures [2,3]. Undergraduate transition to virtual didactics and coursework also hindered the premedical student’s ability to prepare for the MCAT, forcing them to rely on college studies alone. According to Gambril et al. and Lucey et al., this transition likely potentiated existing disparities among students with different financial resources, such as access to costly MCAT preparatory materials [3,4]. Furthermore, the abrupt limit on opportunities to shadow in clinics/hospitals, volunteer in the community, and conduct research created a paucity of regular interpersonal interactions [5,6]. The depth of experiences from which students could participate was therefore limited.

Effect on the medical school admissions process was also glaring, particularly with a shift from in-person to virtual interviews. Anecdotally, the admissions committee at The University of Arizona College of Medicine - Tucson noticed a considerable effect on how to adequately evaluate candidates in a virtual setting. Feedback from students also indicated a degree of heterogeneity in terms of difficulty establishing rapport with interviewers and understanding the school’s culture. To evaluate the change in interview preferences and experiences among medical school candidates, we conducted a comparative retrospective survey of admitted students at a single allopathic medical school.

## Materials and methods

The present analysis was designed as a retrospective cohort study incorporating survey methodology. A 27-question survey was developed to assess the change in interview preferences after the COVID-19 pandemic at The University of Arizona College of Medicine - Tucson (UACOM-T) (Appendix 1). Survey data were collected and managed using the Qualtrics online survey software (Qualtrics XM, Provo, UT, USA). The study was approved by the Institutional Review Board for Human Subjects Research at UACOM-T (Protocol #2107004851). This article was previously posted to the Research Square preprint server on January 3, 2023; however, it is not being considered for full publication elsewhere.

Only admitted and matriculated students to UACOM-T were included in this study. As such, there were two subject groups: the class of 2024 (Co24) and the class of 2025 (Co25). The Co24 had the traditional application experience prior to the pandemic, as medical students from this cohort had primarily in-person interviews (in-person interview group). Responses from the Co24 were therefore used as a baseline control for comparison. The Co25 had applied during the height of the pandemic and were thus limited to primarily virtual interviews (virtual interview group). Regarding dissemination, an email containing the survey link, information about the study, and a copy of IRB approval was sent to the Co24 and Co25 email listservs. To potentially amplify recruitment, gift bags containing UACOM-T gear (e.g., pen, bag, mug) were offered in random drawings for study participants. A response tool indicating completion and thus entry to the random gift drawing allowed for de-identification from the Qualtrics survey, thereby maintaining complete anonymity. Survey responses were collected for three months.

The survey itself was divided into demographic and investigative questions. In the demographics section, students were asked about their age, sex, gender, race, ethnicity, socioeconomic status during childhood, and parent education/occupation. A question about a student’s re-applicant status was also given, thereby allowing a sub-group analysis for this cohort. The investigative questions divided students into the Co24 (in-person interview group) and Co25 (virtual interview group). In this section, students were asked about their undergraduate program, in-state residency, primary and secondary application information (e.g., number of programs applied, cost), CASPer (Computer-based Assessment for Sampling Personal characteristics) test information, interview experiences (e.g., number of invitations, cost, in-person versus virtual versus hybrid formats), and acceptance information. Furthermore, survey participants were also asked about their levels of impression pertaining to the atmosphere/culture of a program, student body, faculty members, curriculum, hospital, housing options, and surrounding neighborhood when in in-person interviews and/or virtual interviews.

Statistical analysis

Quantitative data from Qualtrics were collected into a customized Excel spreadsheet (Microsoft, Redmond, WA, USA). From there, data were pooled and stratified based on responses from the Co24 (in-person interview group) and Co25 (virtual interview group) to acquire retrospective cohorts. Participant rates were defined as the number of medical students who responded to the survey (numerator) divided by the total number of medical students in the respective class (denominator). There are 120 medical students in both classes of 2024 and 2025. Comparisons of categorical data were performed in the Statistical Package for Social Sciences (SPSS) version 27.0 (IBM Corp., Armonk, NY, USA) and ATLAS.ti (ATLAS.ti Scientific Software Development GmbH, Berlin, Germany). The chi-square test on the Likert scale was utilized. Statistical significance was set at p < 0.05.

## Results

There was a total of 195 participants in the survey, however, only 166 responded to Q14 (Are you in the class of 2024 or class of 2025?). As such, the data for 29 respondents with missing information to Q14 were removed from formal analysis. The Co24, or the in-person interview group, therefore had 77 student participants (77/120; 64.2% respondent rate), while the Co25, or the virtual interview group, had 89 (89/120; 74.2% respondent rate). Demographic breakdown showed that a greater percentage of students in the virtual group were in-state applicants (83.1% versus 71.4%), attended The University of Arizona as an undergraduate (46.1% versus 40.3%), required fee assistance from the American Medical College Application Service (15.7% versus 11.7%), and grew up in an underserved community (30.3% versus 19.5%) (Table 1). Overall, the in-person interview group in the class of 2024 had 117 of 216 applicants who were accepted also matriculate (117/216 = 54.2% matriculated; 99/216 = 45.8% declined offer). The virtual group in the class of 2025 had 118 of 197 applicants matriculate (118/197 = 60.0% matriculated; 79/197 = 40.0%).

**Table 1:** Demographic breakdown between in-person and virtual interview groups. Class of 2024, in-person interview group; class of 2025, virtual interview group; AMCAS, American Medical College Application Service

	Class of 2024	Class of 2025
Total Number of Respondents	77	89
Number of Male Respondents	29 (37.7%)	37 (41.6%)
Number of Female Respondents	48 (62.3%)	52 (58.4%)
Age Range	20 - 34	20 - 36
Number of Arizona In-State Residents	55 (71.4%)	74 (83.1%)
Attended The University of Arizona as an Undergraduate	31 (40.3%)	41 (46.1%)
Required Fee Assistance from AMCAS	9 (11.7%)	14 (15.7%)
Grew Up in an Underserved Community	15 (19.5%)	27 (30.3%)

In the in-person interview group, 74.9% and 65.1% of students spent more than $500 on primary applications and travel-related costs (e.g., flight, lodging, transportation), respectively. Further, 14.3% of the in-person interview group had to reschedule their MCAT for any reason (e.g., personal matter, family emergency, COVID-19 related). Of the re-schedulers only, 87.0% of the 11 students postponed one time, 5.2% two times, 7.8% three times, and 0% four or five times. It cost 9.1% of the re-schedulers in the in-person interview group more than $250 in rescheduling fees.

**Figure 1:**
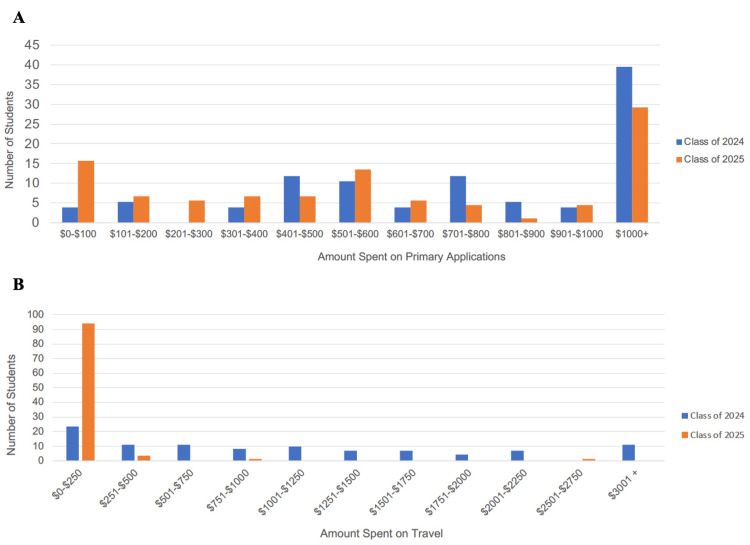
Cost-related differences between medical school applicants prior to (Class of 2024; in-person interview group) and during (Class of 2025; virtual interview group) the COVID-19 pandemic.

In the virtual interview group, 58.4% and 2.4% of students spent more than $500 on primary applications and travel-related costs, respectively (Figure 1A, 1B; p = 0.045 and p < 0.0001; comparison to in-person interview group). Further, 56.1% of the virtual interview group had to reschedule their MCAT for any reason (p < 0.001). Of the re-schedulers only, 48.3% of the 50 students postponed one time, 24.7% two times, 12.4% three times, 12.4% three times, 9.0% four times, and 2.2% five times (p < 0.0001). It cost 24.5% of the re-schedulers at least $250 in rescheduling fees.

**Figure 2:**
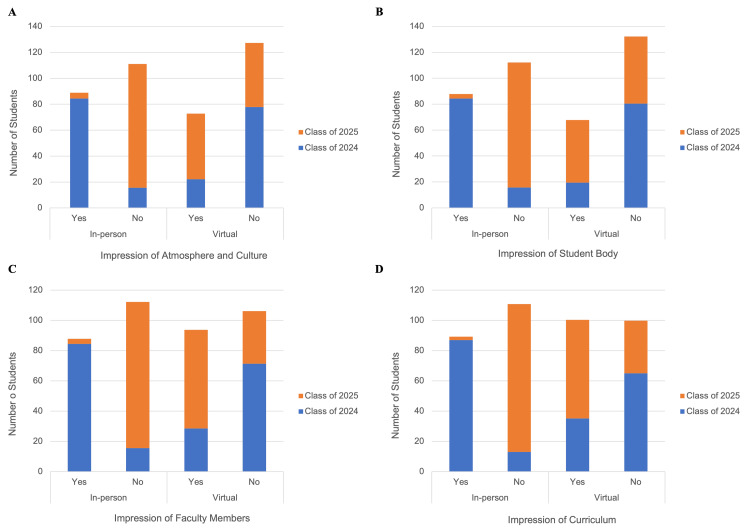
Differences in acquired impressions of a medical school’s atmosphere/culture, student body, faculty, and curriculum between medical school applicants prior to (Class of 2024; in-person interview group) and during (Class of 2025; virtual interview group) the COVID-19 pandemic.

Of the 77 students in the in-person group, 100% preferred an in-person interview format, despite the fact that no student in this cohort had any virtual interviews. Roughly 85.7% of the in-person group would have continued to prefer in-person interviews if presented with either an in-person or virtual format. The in-person group had a greater proportion of students who felt they were able to get a satisfactory impression of the school’s atmosphere/culture (87.0%), student body (75.3%), faculty members (75.3%), curriculum (64.9%), associated hospital(s) (66.2%), housing options (40.3%), and surrounding neighborhoods (63.6%) (Figure 2).

Of the 89 students in the virtual group, 24.1% preferred an in-person interview format (p < 0.0001; comparison to in-person group), despite the fact that only 13.1% of this cohort experienced any in-person interview. The remaining 75.9% preferred the virtual format. Only 22.5% of the virtual group would have preferred an in-person interview if presented with either an in-person or virtual format (p < 0.001). The virtual group, when compared to the in-person group, had a lower proportion of students who felt they were able to get a satisfactory impression of the school’s atmosphere/culture (55.1%), student body (47.2%), faculty members (62.9%), curriculum (61.8%), associated hospital(s) (16.9%), housing options (14.6%), and surrounding neighborhoods (10.1%) (Figure 2; p < 0.001).

## Discussion

According to results from the present survey, the COVID-19 pandemic had a measurable effect on medical school interview preferences and application-related costs from a student’s vantage point. Overall, 85.7% of 77 students in the Co24 (in-person interview group) preferred in-person interviews compared to only 22.5% of 89 students in the Co25 (virtual interview group) (p < 0.001). The shift in these preferences is likely multifactorial. Transition to a virtual setting likely increased the number of interviews that an applicant could attend, thereby increasing the odds of receiving a single acceptance. Further, the affordability of at-home interviews also likely enhanced satisfaction. According to a retrospective survey of applicants to a surgical oncology fellowship during the COVID-19 pandemic, 11/16 (68.8%) respondents expressed a preference for in-person interviews despite a majority (13/16 applicants; 81.3%) feeling able to convey themselves “well” or “very well” over Zoom videoconferences (Zoom Video Communications, San Jose, CA, USA) [7]. Even though a majority preferred live interviews, those applicants highlighted cost and time savings, increased efficiency, and decreased stress-related travel as some benefits to virtual interviewing [7]. The Vining et al. study of surgical oncology fellow applicants included both accepted and rejected candidates, contrasting it from the present study’s narrow inclusion of only accepted students.

It is unclear how many medical schools in the United States transitioned to completely in-person, completely virtual, or hybrid interview formats after the COVID-19 pandemic [8,9]. As such, the findings presented here can potentially guide medical school administrators towards future improvements to the application process. A systematic review conducted by Chandratre and Soman attempted to answer the question of interview style preference for medical school, residency, and/or fellowship entrance regardless of the COVID-19 effect. They found that only 3/15 studies, of which only one was conducted during the COVID-19 era, reported favorability of virtual interviewing [10]. They also found, however, that most applicants (unclear about the percentage) perceived virtual interviews as an impediment to exploring a school’s campus or location, adequately interacting with faculty and peers, and fully expressing their potential as future medical students [10]. Considering that 14/15 survey studies were performed prior to the COVID-19 pandemic, the results from the systematic review should be heeded with caution.

Most of our admitted students from the virtual cohort felt that virtual interviews should be an option going forward for medical school admissions, despite inadequacy in experiencing a school’s culture. This may be related to how virtual interviews can possibly reduce financial barriers for applicants from historically disadvantaged groups, evidenced by how the virtual group here had a greater percentage of students from underserved communities and used the American Medical College Application Service fee waiver, when compared to those students in the in-person group (Table 1). However, this inference should be heeded with caution, as post-COVID-19 loss of jobs and economic burden could impact this finding. Virtual interviews are already a fixture for most medical schools across the United States. However, there most likely will be some hybrid paradigm implemented in the future [11,12]. Possible models have been discussed and include offering a virtual interview as a screening tool followed by an in-person interview or allowing potential candidates to select between the two options [13-15]. The latter choice, though, may foster consternation about applicants being favored implicitly in one interview choice over the other.

Limitations

This study has some limitations. There is an inherent recall bias to retrospective surveys, most likely accentuated in the in-person group responses here since their time away from medical school applications was longer than the virtual group. Future investigators may consider collecting data in real time or immediately after interviews. Further, only matriculated students were surveyed, so preferences cannot be generalized to all applicants, such as those who were not accepted or those who were accepted but chose to attend school elsewhere. There is also a lack of generalizability to admitted medical students at an osteopathic school.

After the COVID-19 pandemic, UACOM-T transitioned to a solely virtual interview experience. However, we recently initiated a “revisit day” for students accepted to the Class of 2026, prior to and independent of a second look day, for applicants to gain a sense of the school’s culture. Possible future directions of this study can thus evaluate the impact of this “revisit day” on applicants’ impressions of a school’s atmosphere.

## Conclusions

The present study found that students who applied to an allopathic medical school during the COVID-19 pandemic had more pre-application hurdles compared to the cohort who applied just prior to the pandemic. Matriculated students who interviewed virtually during the pandemic had less travel-related costs but felt more limited in their experience of a school’s culture and ability to establish rapport with faculty. Despite this, however, the virtual interview group still expressed a preference for the virtual interview format.

## Appendices

**Table 2:** Appendix 1. Copy of Survey Questions

Demographics:																
1. How old were you when you submitted your application to AMCAS? (drop down menu; start at age 18)																
2. Were you a reapplicant? (yes or no)																
3. What do you identify as your sex? (one option)																
a. Female																
b. Male																
c. Prefer not to answer																
4. What do you identify as your gender? (one option)																
a. Female																
b. Gender variant/non-conforming																
c. Male																
d. Transgender																
e. Not listed (free response)																
f. Prefer not to answer																
5. What do you identify as your race/ethnicity? (select all that apply)																
a. American Indian or Alaskan Native																
i. Tribal affiliation (free response)																
b. Asian																
i. Bangladeshi																
ii. Cambodian																
iii. Chinese																
iv. Filipino																
v. Indian																
vi. Indonesian																
vii. Japanese																
viii. Korean																
ix. Laotian																
x. Pakistani																
xi. Taiwanese																
xii. Vietnamese																
xiii. Other Asian (free response)																
c. Black or African-American																
i. African American																
ii. African																
iii. Afro-Caribbean																
iv. Other Black or African American (free response)																
d. Hispanic, Latino, or of Spanish Origin																
i. Argentinean																
ii. Colombian																
iii. Cuban																
iv. Dominican																
v. Mexican/Chicano																
vi. Peruvian																
vii. Puerto Rican																
viii. Other Hispanic, latino, or of Spanish Origin																
e. Native Hawaiian or Other Pacific Islander																
i. Guamanian																
ii. Native Hawaiian																
iii. Samoan																
iv. Other Pacific Islander (free response																
f. White																
g. Other (free response)																
h. Prefer not to answer																
5. Do you identify as any of the following? (select all that apply)																
a. Disadvantaged																
b. Growing up in an underserved community																
c. First-generation college student																
d. First-generation graduate student																
6. Did you receive the Pell grant? (yes or no)																
7. Did you have paid employment before the age of 18? (yes or no)																
8. Do you have military experience? (yes or no)																
9. Is your mother still alive? (yes or no)																
a. Education level (free response)																
b. Occupation (free response)																
10. Is your father still alive? (yes or no)																
a. Education level (free response)																
b. Occupation (free response)																
Investigative Questions:																
12. Were you in the class of 2024 or class of 2025? (one option)																
13. Did you attend The University of Arizona for your undergraduate studies? (yes or no)																
14. Did you have in-state residency in Arizona at the time of your application? (yes or no)																
15. How many schools did you apply to in the primary application? (drop down menu, 1-40+)																
a. Did you receive fee assistance from AMCAS? (yes or no)																
b. How much did you spend on primary applications? (intervals of $100 capping at $1000+)																
c. Did you utilize any paid outside sources for help in compiling your primary application (i.e. workshop, hired freelancers)? (yes or no)																
i. IF YES																
1. Please list and describe the sources you used. (free response)																
2. How much did you spend? (intervals of $100 capping at $1000+)																
16. How many secondary applications did you receive from the schools that you applied to? (drop down menu, 1-40+)																
17. How many secondary applications did you complete? (drop down menu, 1-40+)																
a. Did you receive fee assistance for this? (yes or no)																
b. How much did you spend on secondary applications? (intervals of $100 capping at $3000+)																
c. Did you utilize any paid outside sources for help in compiling your secondary applications (i.e. workshop, hired freelancers)? (yes or no)																
i. IF YES																
1. Please list and describe the sources you used. (free response)																
2. How much did you spend? (intervals of $100 capping at $1000+)																
18. Did you take the CASPer test? (yes or no)																
a. Did you receive fee assistance for this? (yes or no)																
b. How much did you spend on both completing and distributing the CASPer test to schools? (intervals of $10 capping at $100+)																
c. Did you utilize any paid outside sources for help in compiling your secondary applications (i.e. preparation courses)? (yes or no)																
i. IF YES																
1. Please list and describe the sources you used. (free response)																
2. How much did you spend? (intervals of $100 capping at $1000+)																
19. How many interviews did you receive? (drop down menu, 1-20+)																
20. How many interviews did you accept? (drop down menu, 1-20+)																
21. How many interviews did you attend? (drop down menu, 1-20+)																
a. Did you receive fee assistance for this? (yes or no)																
b. How much did you spend on travel-related costs (i.e. flight, lodging, transportation, food)? (intervals of $250 capping at $3000+)																
i. Did you utilize a student host ambassador program? (yes, no, NA)																
c. Did you utilize any paid outside sources for help in preparing for the interview (i.e. preparation courses, hired freelance trainers)? (yes or no)																
i. IF YES																
1. Please list and describe the sources you used. (free response)																
2. How much did you spend? (intervals of $100 capping at $1000+)																
d. What types of interview formats did you experience? (select all that apply)																
i. In-person																
ii. Virtual directly with the school																
iii. Standardized recording format (i.e. AAMC Video Interview Tool)																
iv. Other (free response)																
e. Did any of the interview formats you experienced give you a negative impression of the school? (yes or no)																
i. IF YES																
1. Which ones?																
a. In-person																
b. Virtual directly with the school																
c. Standardized recording format (i.e. AAMC Video Interview Tool)																
d. Other (free response)																
2. Which interview format would you have preferred?																
a. In-person																
b. Virtual directly with the school																
c. Standardized recording format (i.e. AAMC Video Interview Tool)																
d. Other (free response)																
3. How was your experience negative? (optional free response)																
f. Did you experience multiple interview formats? (yes or no)																
i. IF YES																
1. Which one did you prefer?																
a. In-person																
b. Virtual directly with the school																
c. Standardized recording format (i.e. AAMC Video Interview Tool)																
d. Other (free response)																
2. Why? (optional free response)																
g. Did you experience any in-person interviews? (yes or no)																
i. IF YES																
1. Do you think a virtual interview would have positively or negatively impacted your performance when compared to doing it in-person? (positively or negatively)																
2. Do you think a virtual interview would have changed your perspective about the school? (yes or no)																
a. IF YES																
i. How? (optional free response)																
3. Were you able to establish rapport with the interviewer/faculty? (yes or no)																
h. Did you experience any virtual interviews? (yes or no)																
i. IF YES																
1. Do you think an in-person interview would have positively or negatively impacted your performance when compared to doing it virtually? (positively or negatively)																
2. Do you think an in-person interview would have changed your perspective about the school?																
a. IF YES																
i. How? (optional free response)																
3. Were you able to establish rapport with the interviewer/faculty? (yes or no)																
22. How many schools were you accepted to? (drop down menu, 1-10+)																
a. For the school that you made your final decision on (the one you are currently attending or will be attending), did you have an in-person experience? (yes or no)																
i. IF YES																
1. Were you able to get an impression of: (select all that apply)																
a. The atmosphere and culture of the school																
b. The student body																
c. The faculty members																
d. The curriculum																
e. The hospital																
f. The housing options																
g. The surrounding neighborhood																
h. Other (free response)																
2. Do you think that having a virtual experience only would have changed your decision from one school to another? (yes or no)																
a. IF YES																
i. How? (optional free response)																
3. Which format would you have preferred? (select all that apply)																
a. In-person																
b. Virtual																
c. If you selected both, why a mixture? (optional free response)																
b. For the school that you made your final decision on (the one you are currently attending or will be attending), did you only have a virtual experience? (yes or no)																
i. IF YES																
1. Were you able to get an impression of: (select all that apply)																
a. The atmosphere and culture of the school																
b. The student body																
c. The faculty members																
d. The curriculum																
e. The hospital																
f. The housing options																
g. The surrounding neighborhood																
h. Other (free response)																
2. Do you think that an in-person experience would have changed your decision from one school to another? (yes or no)																
a. IF YES																
i. How? (optional free response)																
3. Did you spend free time to visit the school by yourself (i.e. tour the campus, hospital, surrounding neighborhood)? (yes or no)																
a. IF YES																
i. How much did you spend on travel-related costs (i.e. flight, lodging, transportation, food)? (intervals of $250 capping at $3000+)																
4. Which format would you have preferred? (select all that apply)																
a. In-person																
b. Virtual																
c. If you selected both, why a mixture? (optional free response)																
23. How many second looks did you attend? (drop down menu, 1-10+)																
a. Did you receive fee assistance for this? (yes or no)																
b. How much did you spend on travel-related costs (i.e. flight, lodging, transportation, food)? (intervals of $250 capping at $3000+)																
c. Were you able to attend any in-person second looks? (yes or no)																
i. IF YES																
1. Were you able to get an impression of: (select all that apply)																
a. The atmosphere and culture of the school																
b. The student body																
c. The faculty members																
d. The curriculum																
e. The hospital																
f. The housing options																
g. The surrounding neighborhood																
h. Other (free response)																
2. Which format would you have preferred? (select all that apply)																
a. In-person																
b. Virtual																
c. If you selected both, why a mixture? (optional free response)																
d. Were you able to only attend virtual second looks? (yes or no)																
i. IF YES																
1. Were you able to get an impression of: (select all that apply)																
a. The atmosphere and culture of the school																
b. The student body																
c. The faculty members																
d. The curriculum																
e. The hospital																
f. The housing options																
g. The surrounding neighborhood																
h. Other (free response)																
2. Which format would you have preferred? (select all that apply)																
a. In-person																
b. Virtual																
c. If you selected both, why a mixture? (optional free response)																
24. Did you have to reschedule your MCAT for any reason (i.e. personal matter, family emergency, COVID-19)? (yes or no)																
a. IF YES																
i. How many times? (drop down menu, 1-10+)																
ii. Did you receive fee assistance for this? (yes or no)																
iii. How much did you have to spend? (intervals of $250 capping at $1000+)																
iv. Please share other difficulties that you may have had in rescheduling your MCAT, including any other additional costs such as for preparation courses. (optional free response)																
25. Did COVID-19 have an impact on your decision to attend a particular school? (yes or no)																
a. IF YES																
i. Which of the following impacted your decision: (select all that apply)																
1. The school’s COVID-19 response/protocols																
2. The school’s organization																
3. The possibility of delayed clerkships																
4. The curriculum (i.e. in-person classes, online classes, hybrid)																
5. Other (free response)																
ii. Did any of these negatively impact your decision? (yes or no)																
1. IF YES																
a. Which ones?																
i. The school’s COVID-19 response/protocols																
ii. The possibility of delayed clerkships																
iii. The curriculum (i.e. in-person classes, online classes, hybrid)																
iv. Other (free response)																
b. How did it negatively impact your decision? (optional free response)																
26. Do you think you had sufficient information to make an informed decision about the school you are/will be attending? (yes or no)																
a. IF YES																
i. Do you wish you had more time with: (select all that apply)																
1. Learning more about the curriculum																
2. Diversity and inclusion presentation																
3. Medical students																
4. Interviewer																
5. Faculty																
6. Dean																
7. Medical school campus tour																
8. Hospital tour																
9. Other (free response)																
27. Did you feel limited in your ability to learn more about a school? (yes or no)																
a. IF YES																
i. How? (free response)																
